# Therapeutic Efficacy of onabotulinumtoxinA Delivered Using Various Approaches in Sensory Bladder Disorder

**DOI:** 10.3390/toxins12020075

**Published:** 2020-01-23

**Authors:** Po-Yen Chen, Wei-Chia Lee, Hung-Jen Wang, Yao-Chi Chuang

**Affiliations:** 1 Division of Urology, Kaohsiung Chang Gung Memorial Hospital and Chang Gung University College of Medicine, Kaohsiung 83301, Taiwanhujewang@yahoo.com.tw (H.-J.W.); chuang82@ms26.hinet.net (Y.-C.C.); 2 Center for Shock Wave Medicine and Tissue Engineering, Kaohsiung Chang Gung Memorial Hospital, Kaohsiung 83301, Taiwan

**Keywords:** drug delivery, interstitial cystitis, onabotulinumtoxinA, overactive bladder, painful bladder syndrome

## Abstract

Cystoscopic onabotulinumtoxinA (onaBoNTA) intradetrusor injection is an efficient and durable modality for treating sensory bladder disorders. However, the inconvenience of using the cystoscopic technique and anesthesia, and the adverse effects of direct needle injection (e.g., haematuria, pain, and infections) have motivated researchers and clinicians to develop diverse injection-free procedures to improve accessibility and prevent adverse effects. However, determining suitable approaches to transfer onaBoNTA, a large molecular and hydrophilic protein, through the impermeable urothelium to reach therapeutic efficacy remains an unmet medical need. Researchers have provided potential solutions in three categories: To disrupt the barrier of the urothelium (e.g., protamine sulfate), to increase the permeability of the urothelium (e.g., electromotive drug delivery and low-energy shock wave), and to create a carrier for transportation (e.g., liposomes, thermosensitive hydrogel, and hyaluronan-phosphatidylethanolamine). Thus far, most of these novel administration techniques have not been well established in their long-term efficacy; therefore, additional clinical trials are warranted to validate the therapeutic efficacy and durability of these techniques. Finally, researchers may make progress with new combinations or biomaterials to change clinical practices in the future.

## 1. Introduction

Since the first injection of onabotulinumtoxinA (onaBoNTA) for a patient with detrusor sphincter dyssynergia in 1988, onaBoNTA has been extensively used in the treatment of lower urinary tract dysfunction [1]. At present, the intradetrusor injection of onaBoNTA is indicated for treating neurogenic and idiopathic overactive bladder (OAB) [2]. Although the intradetrusor injection of onaBoNTA is highly efficacious in the treatment of OAB and other sensory bladder disorders, some perioperative complications (e.g., pain, haematuria, increased post-void residual volume, acute urinary retention, and urinary tract infections) and inconvenience related to performing the cystoscopic procedure along with anesthesia, remain a concern [3]. Hence, researchers have attempted to deliver onaBoNTA by using different approaches to improve accessibility and decrease adverse effects in patients. This review examines these experimental studies of injection-free onaBoNTA delivery for the treatment of sensory bladder disorders.

## 2. Sensory Bladder Disorders

**Figure 1:**
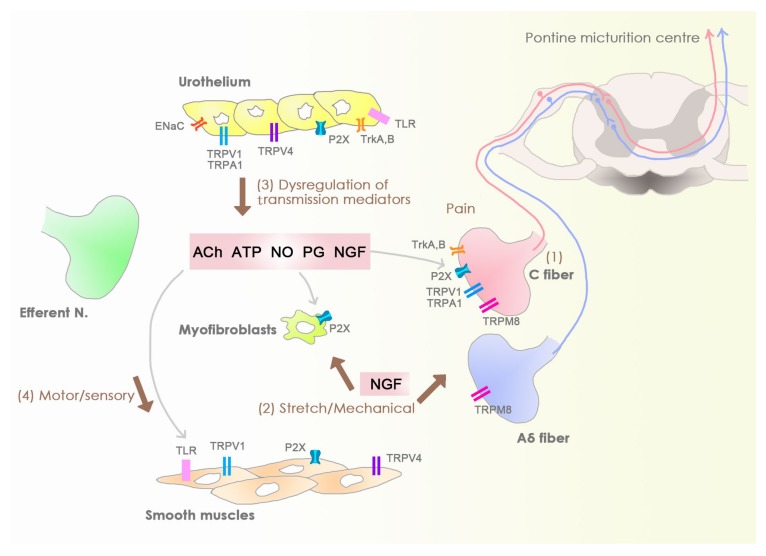
Sensory bladder disorders: The sensory disturbances of an unstable bladder may elicit from activation of silence C-fibers due to noxious stimuli (1), stretching stretch-mediated receptors, and myofibroblasts (2), chemicals inducing urothelial signaling (3), and signals generated in a modulated motor-sensory system (4).

Sensory bladder disorders are caused by sensation abnormality and include OAB syndrome and interstitial cystitis/painful bladder syndrome (IC/PBS). Both of these have similar irritative bladder symptoms but have different clinical features in terms of urinary incontinence and bladder pain. In OAB patients, urgency is the cardinal symptom along with urinary frequency, nocturia, and urgent incontinence. By contrast, the patients with (IC/PBS) are bothered by bladder pain, urinary frequency, and nocturia [4]. The brief description of probable sensory disturbances occurrence are illustrated in Figure 1.

The pathophysiology of OAB is not well understood but may involve disorders of urothelial signaling, detrusor muscle instability, and nerve hyperexcitability [5]. For instance, metabolic derangement, bladder outlet obstruction, and inflammation can underlie the disturbances of these three factors of the bladder and can contribute to OAB. OAB has a high prevalence (around 16%–20%) [6], and its symptoms have detrimental effects on individual quality of life and incur societal costs for public health [7]. IC/PBS may develop due to urothelial dysfunction and cause an inflammatory reaction of the bladder and cause permanent inflammatory imprinting in the central nervous system [4]. Dysfunction of the urothelial lining may cause the activation of mast cells and the winding up of capsaicin-sensitive nerves. The release of neurotransmitters from the urothelium and nerve endings then provokes peripheral and central pain sensitization. The prevalence of IC/PBS among adult women is estimated to be between 2.7% and 6.5% [8].

The first-line therapies for OAB patients are behavioral therapies and lifestyle modifications. The second-line treatments include the monotherapy or combinations of antimuscarinics and the β3-adrenoceptor agonist. For refractory cases of OAB, intradetrusor injection of onaBoNTA or neuromodulation can be considered as third-line therapies [9].

For patients with IC/PBS, patient education and behavioral modification are essential in first-line therapy. The oral medications of amitriptyline, cimetidine, hydroxyzine, or pentosan polysulphate as well as the intravesical treatments of dimethyl sulphoxide (DMSO), heparin, or lidocaine are considered as second-line therapies. The third-line treatments include cystoscopic low-pressure hydrodistention under anesthesia and fulguration for Hunner’s lesions. Intradetrusor injection of onaBoNTA is the fourth-line therapy for selective IC/PBS patients [10].

## 3. Mechanism of Action of onaBoNTA

OnaBoNTA, a botulinumtoxinA with the most well-understood effects of therapy, is used in clinical settings. It is a neurotoxic protein that can bind its 100 kDa heavy chain to synaptic vesicle protein 2 as its protein receptor and enter the neuron endings; thereafter, its 50 kDa light chain is released, translocated to the cytosol [2,3]. The light chain of onaBoNTA cleaves synaptosomal nerve-associated protein (SNAP) 25, blocks soluble N-ethylmaleimide-sensitive fusion attachment protein receptor complex, and interferes with synaptic acetylcholine vesicle fusion to the cell membrane from presynaptic efferent nerves; thus, it causes paralysis of the detrusor muscle and modulation of the postsynaptic receptors. Furthermore, onaBoNTA intradetrusor injection possesses the ability to interrupt C-fibre sensory transmission, block the release of neurotransmitters (e.g., substance P, adenosine triphosphate, and calcitonin gene-related peptide), prevent receptor trafficking (TRPV1 as an example), and decrease ATP and nerve growth factor (NGF) releasing and affects preganglionic parasympathetic nerve terminals [11]. Due to its chemodenervation effects, onaBoNTA may provide anti-inflammation and antinociceptive effects for patients against neurogenic inflammation along with blocking the release of neuropeptides [3].

## 4. Barriers and Sensory Web of the Bladder Mucosa and Submucosa

The bladder wall has four layers: Mucosa, submucosa (lamina propria and muscularis mucosae), detrusor muscle, and serous layer (tunica serosa) [12]. The transitional urothelium extends from the renal pelvis to the ureter and inner bladder wall. The urothelial apical surface lines a sulfated polysaccharide glycosaminoglycan layer, which acts as a non-specific anti-adherence factor and as a defense mechanism against infection. The urothelium is composed of three layers: A basal cell layer, an intermediate layer, and a superficial layer containing polarized umbrella cells (diameters of 25–250 μm). The uroplakin membrane and tight junction complexes of the umbrella cell layer play important roles in the barrier function of the urothelium. The apical uroplakin membrane may reduce the permeability of the cells to small molecules (e.g., water, urea, and protons). The tight junction complexes can reduce the movement of ions and solutes between cells and specialized lipid molecules. The intermediate spindle cells, found beneath the umbrella layer, have up to five strata, and they can rapidly differentiate into umbrella cells when the barrier is disrupted. The single-layer mononuclear basal cells adhere to the intermediate cells and to the basement membrane. The suburothelial layer consists of interstitial cells, myofibroblasts, blood vessels, and afferent sensory nerve endings at the lamina propria beneath the basal membrane.

**Figure 2:**
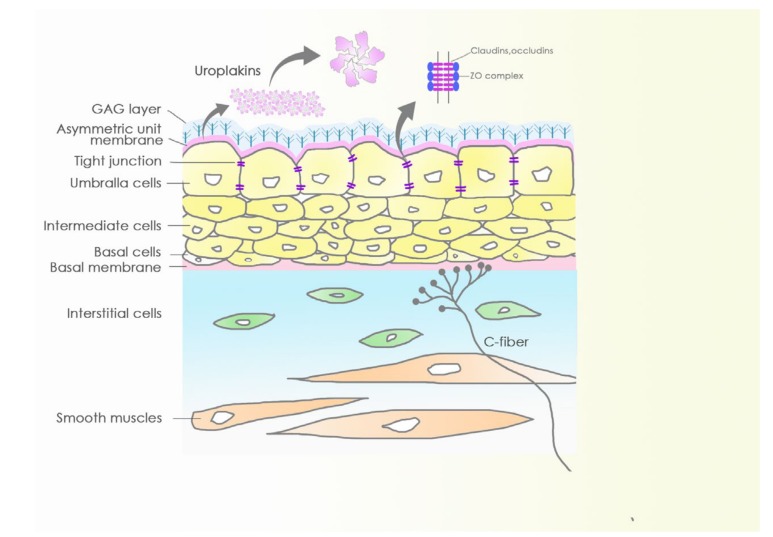
Schematic diagram of barriers and sensory web of the bladder mucosa. Impermissible bladder barrier consists of the glycosaminoglycan (GAG) layer, uroplakins, and tight junction. The sensory web was innervated by neuron-like urothelium.

In addition to the barrier function, the mucosa layer serves as a sensory web, which comprises the urothelium and sensory afferent and efferent nerves linked by gap junctions, which amplify and transmit the signals among the mucosa, nervous, and muscular systems [3,12]. The unmyelinated C-fiber endings are widespread in the urothelium and lamina propria. Parasympathetic nerve cells and intramural ganglion cells are also embedded among the lamina propria. The schematic diagram of the bladder mucosa and submucosa are illustrated in Figure 2.

## 5. Intravesical Delivery of onaBoNTA

### 5.1. Passive Diffusion

**Figure 3:**
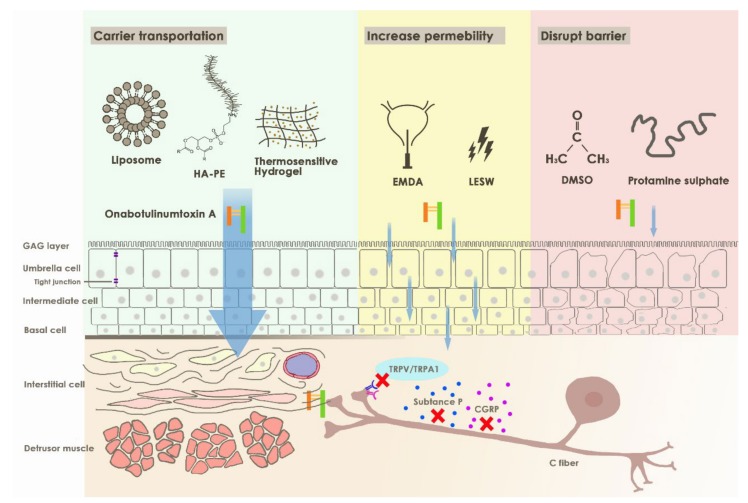
Illustration of different approaches for onabotulinumtoxinA delivery in sensory bladder disorder.

In an animal study, Khera et al. reported that onaBoNTA failed to reach the muscle layer by direct bladder instillation. Another report from Coelho et al. further supported the lack of effect of onaBoNTA when simply instilled in the bladder with an intact urothelium [13,14]. Bladder instillation of onaBoNTA was less efficient owing to multiple factors. First, onaBoNTA is a large molecule (150 KDa). For targeting the suburothelium and detrusor muscle, passive diffusion is limited by tight junctions [15]. Second, the onaBoNTA agents might be diluted and degraded by urine proteases due to daily urine production of 800 to 2000 mL. Therefore, urologists need a modality that can cross the urothelium barrier, penetrate deep into the bladder, and persist for a sufficient time. Some studies have attempted to overcome the problems with different approaches, as illustrated in Figure 3.

### 5.2. Disrupt Barrier

#### 5.2.1. Protamine Sulfate

Protamine sulfate, an arginine-rich protein, can increase permeability in the apical membrane to both cations and anions by altering the membrane conductance in different concentrations and by damage to the surface of the bladder mucosa [16,17]. In addition, protamine sulfate also has analgesic effects, tissue-protective effects, and pro-inflammatory response suppression for relieving bladder pain in mice [18].

In a study, rats with spinal cord injuries received 1 mL of 1% protamine sulfate bladder instillation, followed by 1 mL of onaBoNTA (20 U). By using this procedure, Khera et al. reported that onaBoNTA could inhibit vesical sensory mechanisms but could not alter bladder motor function in rats. These results denoted that onaBoNTA could not penetrate across the urothelium into detrusor muscle, even after the urothelium was disrupted by protamine sulfate instillation [13].

#### 5.2.2. Dimethyl Sulphoxide

Dimethyl sulphoxide (DMSO) intravesical instillation was developed some decades ago by Stewart et al. [19]. Now, DMSO is an FDA-approved second-line therapy for IC/BPS [20]. DMSO, an organic solvent, can desquamate urothelium, disrupt mucosa, and interfere with cellular phospholipid membranes. In addition, DMSO luminal effluent leads to a loss of urothelial layers and mucosal folding; thus, it facilitates membrane penetration to increase urothelium permeability and to cause leakage of cytosolic contents. Besides, DMSO was found to inhibit the stretch-activated adenosine triphosphate release from urothelial cells and relax the detrusor muscle in rabbits [21].

Petrou et al. conducted a phase 1/2 study in treating refractory idiopathic detrusor overactivity in women by using 300 U of onaBoNTA plus 50% DMSO solution for instillation [22]. The results demonstrated that patients who underwent this treatment had improved incontinence events, decreased Indevus Urgency Severity Scale scores, and improved quality of life as recorded by questionnaires (i.e., 6-item Urogenital Distress Inventory, bothersome score, and Incontinence Impact Questionnaire short-form) at the endpoint of the 3-month observation.

### 5.3. Increase Permebility

#### 5.3.1. Electromotive Drug Administration

Electromotive drug administration (EMDA) was designed in 1996 to transfer drugs into the bladder submucosa area for treating bladder cancer [23]. The previous review mentioned that EMDA contained three phenomena: Iontophoresis, electro-osmosis, and electroporation. Iontophoresis passes an electrical current forming the active charged ingredient and propels a substance into tissues. Electro-osmosis transfers non-ionized polar molecules and ionized molecules against their coulombic gradients. And electroporation increases electrical conductivity and permeability of the cell plasma membranes [24]. In clinical trials, electromotive delivery of mitomycin has been used to treat non–muscle-invasive bladder cancer to reduce its recurrence rate [25,26]. A basic study also reported that EMDA systems could enhance mitomycin transport into the full-thickness of the bladder wall without chemical modification or without changing the histology of the urothelium, lamina propria, and muscularis [27]. Nowadays, some studies apply EMDA on improving local anesthesia effects and the treatment of detrusor overactivity and interstitial cystitis in patients. The drugs under EMDA delivery studies include lidocaine, mitomycin C, oxybutynin, verapamil, resiniferatoxin, and dexamethasone [28].

Researchers applied EMDA to transfer onaBoNTA into the bladder of children with myelomeningocele-associated neurogenic bladder. They instilled onaBoNTA solution into children’s bladders and delivered 10 mA of pulsed currents for 15 or 20 min without anesthesia [29,30]. The results of these studies demonstrated that children with myelomeningocele-associated neurogenic bladder might benefit from this administration in terms of urinary incontinence and vesicoureteral reflux. Because these studies lacked a control group, the treatment benefit in other sensory disorders remains unclear.

#### 5.3.2. Low-Energy Shock Wave

The term “shock wave” indicates a high-energy sound wave that terminates in a burst of energy, similar to a mini-explosion. Shock waves, continuously transmitted sonic waves at a 16–20 MHz frequency, can carry energy from an area of positive pressure to an area of negative pressure to propagate through mediums. Shock waves (SWs) may be applied in various medical situations because of its unique physical, physical–chemical, chemical, and biological effects. Thus, LESWs per se might have therapeutic effects for inflammatory disorders, such as chronic prostatitis and cystitis. In the physical phase, the tensile force of SW creates negative pressure, which causes cavitation and molecule ionization to affect the permeability of the plasma membrane. In basic studies, high energy levels (>0.12 mJ/mm^2^) SWs altered cell structure and organelles. By contrast, the capability of low-energy shock waves was to increase the tissue permeability without consequent cytotoxicity temporarily; thus, LESWs can facilitate the transfer of pharmaceutical molecules into cells [31].

Through LESW induction, Chuang et al. recorded bladder urothelium leakage of Gd-diethylenetriamine pentaacetic contrast medium through magnetic resonance imaging in rats [32]. Under these circumstances, intravesical onaBoNTA can penetrate into the bladder and suppress the rat’s bladder hyperactivity induced by acetic acid. Nageib et al. conducted a clinical study to prove this concept of injection-free onaBoNTA delivery through LESWs [33]. They recruited 15 refractory OAB patients who received 100 U of onaBoNTA bladder instillation along with LESWs consisting of 3000 shocks over 10 min. The study results revealed a significant decrease in OAB symptom scores in patients at the 1- and 2-month checkpoints, but not at 3 months.

### 5.4. Carrier Transportation

#### 5.4.1. Liposome Formulation of onaBoNTA

Liposomes are spherical lipid vesicles composed of phospholipid bilayers surrounded by an aqueous core. Liposomes can incorporate pharmaceutical drugs, both hydrophilic and hydrophobic, and transfer various sizes of drug molecules through the urothelium via the endocytosis mechanism [34]. Moreover, liposomes can coat the urothelium and assist in its repair in case of injury. Thus, only empty liposome bladder instillation can ameliorate the urinary symptoms and pain scale of patients with IC/PBS [35].

Chuang et al. examined the effects of liposome formulation of onaBoNTA (hereafter, lipotoxin) in a rat model [36]. The results demonstrated that intravesical lipotoxin instillation could cleave SNAP-25, inhibit calcitonin gene-related peptide release from afferents, and suppress bladder hyperactivity induced by acetic acid. Furthermore, Kuo et al. reported that bladder instillation of lipotoxin could reduce the urinary frequency at 1 month in OAB patients [37]. In a subsequent clinical trial, Chuang et al. recruited 62 participants with OAB and demonstrated that a single intravesical instillation of lipotoxin did work at 4 weeks by improving urgency but not beyond that time point [38].

Researchers are also considering the application of lipotoxin for IC/BPS or other bladder sensory disorders. However, Chuang and Kuo demonstrated that the therapeutic effect of a single intravesical instillation of lipotoxin for patients with IC/PBS would be similar to the placebo effect [39]. Recently, Lee et al. investigated the effects of lipotoxin on ketamine-induced ulcerative cystitis in rats [40]. The results showed repeated lipotoxin bladder instillation could protect the urothelium against the insults of urinary ketamine and its metabolites and restore the urothelial tight junction and adhesion proteins (i.e., zonula occludens-1 and E-cadherin). The chemodenervation effect of onaBoNTA could also be observed in this experiment, including the modulation of the detrusor M2 receptor, suppression of the neurogenic inflammation processes, and reduction in immune reactions. Hence, further studies validating the long-term effects of lipotoxin in single or multiple instillations in treating sensory bladder disorders are warranted.

#### 5.4.2. Intravesical Thermosensitive Hydrogel

Thermosensitive hydrogel consists of PEG-PLGA-PEG triblock copolymers, which can provide a composition to slowly release low-molecular-weight hydrophobic drugs lasting for 2 months [41]. The thermosensitive hydrogel exists in a liquid state at room temperature (25 °C) or below, but converts to a semisolid state at elevated temperatures, such as body temperature (37 °C) [42]. After the hydrogel is instilled into the bladder, it can act as a matrix filled drug for maintaining a prolonged exposure of drugs at the urothelium.

For treating patients with idiopathic OAB, Krhut et al. conducted a 1-month clinical trial using onaBoNTA embedded in an inert hydrogel [43]. The results revealed that intravesical instillation of 200U onaBoNTA, embedded in TC-3 gel, might have a therapeutic benefit for some patients with OAB. In a pilot study, Rappaport et al. used onaBoNTA embedded in TC-3 gel to treat patients with IC/PBS and reported a borderline therapeutic effect for participants on the visual analog scale at week 12 [44].

#### 5.4.3. Hyaluronan-Phosphatidylethanolamine

Hyaluronic acid, also called hyaluronan (HA), is a hydrophilic polysaccharide and a common ingredient in skincare products for its epidermis-healing function. A nonparticulate formula was developed by linking HA to phosphatidylethanolamine (PE), which increases HA levels through epidermal cell layers. This high viscosity formulation of HA-PE could be applied as a carrier for transferring large proteins, such as onaBoNTA, through the urothelium. In a rat model, Shatoury et al. proved that bladder instillation of onaBoNTA enmeshed in HA-PE could transfer onaBoNTA through bladder mucosa. According to the study, both routes of HA-PE and intradetrusor injections had comparable SNAP-25 cleavage [45].

## 6. Conclusions

In this review, the suitable modalities for transferring onaBoNTA into the bladder were discussed. The summary of therapeutic effects in clinical trials among various drug delivery systems is illustrated in Table 1. The efficacy of current solutions did not reach a similar efficacy as intradetrusor injections. In sum, passive diffusion is less effective and practical in clinical use. Protamine sulfate failed to deliver the onaBoNTA into the detrusor layer of the bladder, even in an animal model. Using EMDA to conduct onaBoNTA into the bladder seems promising in treating neurogenic OAB in children. It resulted in a 9-month decrease of detrusor pressure and alleviated urinary incontinence episodes and vesicoureteral reflux. LESWs can help the onaBoNTA penetrate into the bladder and maintain its therapeutic effects for 2 months in a clinical trial. Using DMSO as a delivery agent of onaBoNTA may benefit patients with idiopathic detrusor overactivity by improving urinary symptoms and quality of life for 1–3 months. The concept of liposome-encapsulated onaBoNTA has been proven in rat models. However, in clinical trials, the current lipotoxin regimen can only improve the OAB symptoms of patients for 1 month. At present, a thermosensitive hydrogel is also under clinical trial for treating urothelial cancer by embedding mitomycin. In terms of treating OAB or IC/PBS, the therapeutic effect of thermosensitive hydrogel embedded with onaBoNTA is barely satisfactory. HA-PE, a novel agent, can carry onaBoNTA across the urothelium of rats’ bladders. Additional clinical trials are needed to prove its efficacy. It is worth noting that most of the literatures in this review did not report serious adverse effects during the studies. It may attribute to the small sample size and single arm studies thus that determining causes and unexpected adverse events might be unnoticed. The needle-free delivery techniques of onaBoNTA through bladder instillation should continuously be improved for treating sensory bladder disorders, and the safety and efficacy are worthy of being further investigated.

## Figures and Tables

**Table 1:** Clincal trials for sensory bladder disorders with onabotulinumtoxinA injection-free procedures.

Research and Modalities	No.Pts	Study Design	Follow-up Duration	Patients Criteria	Modalities Utilization and onaBoNTA Dose	Outcome at the End Point
Petrou et al. [22] (DMSO)	25	Single arm Prospective cohort	3 months	Adult females idiopathic DO	DMSO (50% 50mL) plus 300U onaBoNTA	Improve incontinence at 1 month, but not at 3 months
Kajbafzadeh et al. [29] (EMDA)	15	Single arm Prospective cohort	9 months	Children MMC-related DO	onaBoNTA (10 U/kg) plus EDMA delivered 10 mA for 15 min	Improve incontinence in 80% cases and decrease VUR grade in 58% cases
Kajbafzadeh et al. [30] (EMDA)	24	Single arm Prospective cohort	6 years	Children MMC-related DO	onaBoNTA (10 U/kg) plus EDMA delivered 10 mA for 20 min	Followed up at 1, 2, 3, 5, 6 years, and 75%, 45.5%, 37.5%, 33%, 29.1% of patients remain completely dry
Nageib et al. [33] (LESW)	15	Single arm Prospective cohort	3 months	Adults idiopathic DO	100U onaBoNTA plus LESW 3000 shocks (6.6 mJ/shock, 300 shocks/min)	Improve OABSS at 1, 2 months, but not at 3 months.
Kuo et al. [37] (Liposome)	24	Double-blind RCT	3 months	Adults OAB	200U onaBoNTA plus 80 mg liposomes	Improve frequency at 1 month, but not at 3 months
Chuang et al. [38] (Liposome)	62	Multicenter double-blind RCT	4 weeks	Adults OAB	200U onaBoNTA plus 80 mg liposomes	Decrease micturition events and urgency severity at 4 weeks
Chuang and Kuo [39] (Liposome)	96	Multicenter double-blind RCT	4 weeks	Adults IC/BPS	200U onaBoNTA plus 80 mg liposomes	Improve OSS, ICSI, ICPI, VAS scores, but not superior to placebo at 4 weeks
Krhut et al. [43] (Hydrogel)	39	Double-blind RCT	1 month	Adult females OAB	200U onaBoNTA plus TC-3 gel	Improve urgency, leakage episodes, PPBC, OAB-q scores at 1 month
Rappaport et al. [44] (Hydrogel)	15	Single arm Prospective cohort	12 weeks	IC/BPS	200IU onaBoNTA plus 40 mL TC-3 Gel	Improve ICSI, VAS scores at week 12

MMC, myelomeningocele; DO, detrusor overactivity; OSS, O’Leary-Sant symptom scores; IC/BPS, interstitial cystitis/bladder pain syndrome; ICSI, ICPI, Interstitial cystitis symptom, and problem indices; VAS, visual analog scale; PPBC, Patient Perception of Bladder Condition; OAB-q, Overactive Bladder Questionnaire; FBC, functional bladder capacity; GRA, global response assessment.
